# Gene Expression Profiling of Transcription Factors of *Helicobacter pylori* under Different Environmental Conditions

**DOI:** 10.3389/fmicb.2017.00615

**Published:** 2017-04-10

**Authors:** Miguel A. De la Cruz, Miguel A. Ares, Kristine von Bargen, Leonardo G. Panunzi, Jessica Martínez-Cruz, Hilda A. Valdez-Salazar, César Jiménez-Galicia, Javier Torres

**Affiliations:** ^1^Unidad de Investigación Médica en Enfermedades Infecciosas y Parasitarias, Hospital de Pediatria, Centro Médico Nacional Siglo XXI, Instituto Mexicano del Seguro SocialMexico City, Mexico; ^2^Phico Therapeutics LtdCambridge, UK; ^3^CNRS UMR7280, Inserm, U1104, Centre d’Immunologie de Marseille-Luminy, Aix Marseille Université UM2Marseille, France; ^4^Laboratorio Clínico, Unidad Médica de Alta Especialidad, Hospital de Pediatría, Centro Médico Nacional Siglo XXI, Instituto Mexicano del Seguro SocialMexico City, Mexico

**Keywords:** *H. pylori*, transcription factors, environmental cues

## Abstract

*Helicobacter pylori* is a Gram-negative bacterium that colonizes the human gastric mucosa and causes peptic ulcers and gastric carcinoma. *H. pylori* strain 26695 has a small genome (1.67 Mb), which codes for few known transcriptional regulators that control bacterial metabolism and virulence. We analyzed by qRT-PCR the expression of 16 transcriptional regulators in *H. pylori* 26695, including the three sigma factors under different environmental conditions. When bacteria were exposed to acidic pH, urea, nickel, or iron, the sigma factors were differentially expressed with a particularly strong induction of *fliA*. The regulatory genes *hrcA, hup*, and *crdR* were highly induced in the presence of urea, nickel, and iron. In terms of biofilm formation *fliA, flgR, hp1021, fur, nikR*, and *crdR* were induced in sessile bacteria. Transcriptional expression levels of *rpoD, flgR, hspR, hp1043*, and *cheY* were increased in contact with AGS epithelial cells. Kanamycin, chloramphenicol, and tetracycline increased or decreased expression of regulatory genes, showing that these antibiotics affect the transcription of *H. pylori*. Our data indicate that environmental cues which may be present in the human stomach modulate *H. pylori* transcription.

## Introduction

*Helicobacter pylori* is a Gram-negative bacterium, a member of the Epsilon proteobacteria that colonizes the human gastric mucosa and is responsible for causing peptic ulcers and gastric carcinoma (Marshall and Warren, 1984; Parsonnet et al., 1991; Uemura et al., 2001). *H. pylori* survives in the hostile environment found in the stomach, which is partially attributed to the expression of virulence factors, such as secretion systems, cytotoxins, flagella, and adhesins. Unlike other Gram-negative bacteria such as *Escherichia coli* or *Salmonella enterica*, the *H. pylori* genome encodes only few known transcriptional regulators, which control expression of genes involved in bacterial metabolism and pathogenicity. This limited repertoire is likely due to its life style highly adapted to one particular niche, the human gastric mucosa. *H. pylori* strain 26695 has a small genome of 1.67 Mb (Tomb et al., 1997), and possesses three genes that code for sigma factors: *rpoD* (σ^80^), *rpoN* (σ^54^), and *fliA* (σ^28^). σ^80^ is a homolog of Gram-negative vegetative sigma factors responsible for the transcription of housekeeping genes (Tomb et al., 1997; Beier et al., 1998), whereas σ^54^ and σ^28^ are two alternative sigma factors dedicated mostly to control expression of flagella components (Fujinaga et al., 2001; Josenhans et al., 2002; Niehus et al., 2004). The response regulator FlgR is also involved in regulation of flagella synthesis (Spohn and Scarlato, 1999b), whereas bacterial chemotaxis is controlled by the CheY protein (Foynes et al., 2000; Terry et al., 2005). Master regulators of response to metals such as Fur, NikR, and CrdR activate or repress genes in the presence of iron, nickel or copper, respectively (Contreras et al., 2003; Waidner et al., 2005; Pich and Merrell, 2013). Environmental cues such as acid pH and temperature influence expression of HrcA, HspR, and ArsR regulatory proteins (Spohn and Scarlato, 1999a; Spohn et al., 2004; Pflock et al., 2005). Although many microarrays analysis have been published (Ang et al., 2001; Merrell et al., 2003a,b; Thompson et al., 2003; Wen et al., 2003; Bury-Mone et al., 2004; Kim et al., 2004), little is known about the effects of environmental cues on the expression of *H. pylori* regulatory genes, including some poorly investigated transcriptional regulators.

In this work, we determined the expression profile of the transcriptional repertoire of *H. pylori* strain 26695 under several environmental conditions relevant for adaptation to its particular ecological niche of the human stomach, such as acidic pH, urea, nickel, and iron. In addition, we analyzed the expression of regulatory genes in biofilm formation and in the presence of AGS gastric epithelial cells. Finally, we studied the effect of the antibiotics kanamycin, chloramphenicol, and tetracycline on the transcription of regulatory genes. Our study describes the transcriptional expression of *H. pylori* regulatory genes in response to different environmental conditions.

## Materials and Methods

### *In silico* Identification of *H. pylori* Transcription Factors

Selection of *H. pylori* transcription factors was performed as previously reported in the literature [(see **Table 1**) (Danielli et al., 2010)]. Sequence data and loci annotations from 260 *H. pylori* genomes were retrieved from the NCBI database^1^ by a series of custom Perl scripts. In addition, the genomes of 57 *Helicobacter* non-*pylori* strains were included in the comparative analysis (Supplementary Table S1). Each putative transcriptional factor was queried using PSI-BLAST (Altschul et al., 1997) under the following parameters: matrix = BLOSUM62, word size = 3, PSI-BLAST threshold = 0.005, expect threshold = 10, and without filtering low complexity regions. Hits were carefully examined and selected according to their functional annotation.

**Table 1:** Transcription factors of *Helicobacter pylori* 26695.

Gene	Protein/Reference sequence	Functions	Reference
*rpoD* (*hp0088*)	RpoD, σ^80^/NP_206888.1	Vegetative sigma factor, transcription of housekeeping genes	Tomb et al., 1997; Beier et al., 1998
*rpoN* (*hp0714*)	RpoN, σ^54^/NP_207508.1	Alternative sigma factor, expression of class II flagellar genes, stress and virulence	Josenhans et al., 2002; Niehus et al., 2004; Sun et al., 2013
*fliA* (*hp1032*)	FliA, σ^28^/NP_207822.1	Alternative sigma factor, expression of class III flagellar genes, stress and virulence	Josenhans et al., 2002; Niehus et al., 2004; Baidya et al., 2015
*hrcA* (*hp0111*)	HrcA/NP_206911.1	Involved in heat shock, stress response and motility	Spohn et al., 2004; Roncarati et al., 2007, 2014
*arsR* (*hp0166*)	ArsR/NP_206965.1	Acid adaptation, acetone metabolism, oxidative stress response, quorum sensing, and biofilm formation	Pflock et al., 2005, 2006; Loh et al., 2010; Servetas et al., 2016
*hp0222*	HP0222/NP_207020.1	Possibly involved in acid response and/or bacterium-epithelial cell contact	Ang et al., 2001; Kim et al., 2004; Popescu et al., 2005
*hp0564*	HP0564/NP_207359.2	Paralog of HP0222; unknown function	Borin and Krezel, 2008
*flgR* (*hp0703*)	FlgR/NP_207497.1	RpoN-dependent master regulator of class II flagellar genes	Spohn and Scarlato, 1999b; Brahmachary et al., 2004
*hup* (*hp0835*)	HU/NP_207628.1	Acid stress response, DNA protection from oxidative stress, involved in mouse colonization	Almarza et al., 2015; Wang and Maier, 2015
*hp1021*	HP1021/NP_207811.1	Chromosomal replication regulator, acetone metabolism and growth	Beier and Frank, 2000; Pflock et al., 2007a; Donczew et al., 2015
*hspR* (*hp1025*)	HspR/NP_207815.1	Heat shock, acidic/osmotic stress response, and motility	Spohn and Scarlato, 1999a,b; Spohn et al., 2002, 2004; Roncarati et al., 2007
*fur* (*hp1027*)	Fur/NP_207817.1	Pleiotropic regulator involved in acid adaptation, metal homeostasis and Mongolian gerbil/mouse colonization	Bijlsma et al., 2002; Harris et al., 2002; van Vliet et al., 2003; Bury-Mone et al., 2004; Danielli et al., 2006; Gancz et al., 2006
*hsrA* (*hp1043*)	HsrA/NP_207833.1	Cell viability, oxidative stress, virulence, response to metronidazole	Olekhnovich et al., 2013, 2014; Vannini et al., 2016
*cheY* (*hp1067*)	CheY/NP_207858.1	Chemotaxis, motility, Mongolian gerbil/mouse colonization	Beier et al., 1997; Foynes et al., 2000; McGee et al., 2005; Terry et al., 2005
*nikR* (*hp1338*)	NikR/NP_208130.1	Nickel-response pleiotropic regulator, metal (copper, iron, and nickel) homeostasis, acid adaptation, iron uptake/storage, motility, chemotaxis, stress response, and mouse colonization	Contreras et al., 2003; Bury-Mone et al., 2004; Ernst et al., 2005b; Muller et al., 2011
*crdR* (*hp1365*)	CrdR/NP_208157.1	Copper resistance, survival under nitrosative stress, and mouse colonization	Panthel et al., 2003; Waidner et al., 2005; Hung et al., 2015

### Bacterial Strains and Culture Conditions

*H. pylori* 26695 was grown for 3 days on blood agar plates containing 10% defibrinated sheep blood, at 37°C under microaerophilic conditions. A bacterial suspension was prepared in *Brucella* broth (BB), and adjusted to an optical density of 0.1 at 600 nm (2 × 10^6^ CFU/ml). *H. pylori* was then grown at 37°C for 24 h (logarithmic growth phase) or 48 h (stationary growth phase) in BB supplemented with 10% decomplemented fetal bovine serum (BB + FBS) under the following conditions: adjusted to pH 5.5, or containing either urea [5 mM CO(NH_2_)_2_], nickel [250 mM NiCl_2_], or iron [150 mM (NH_4_)_2_Fe(SO_4_)_2_⋅6H_2_O] as previously described (Contreras et al., 2003; Wen et al., 2003; Vannini et al., 2014; Cardenas-Mondragon et al., 2016). Fold-changes in transcription were determined by calculating the relative expression of transcription regulator genes under different environmental conditions as compared to expression in BB + FBS. Experiments were performed in triplicate on three different days and the results shown are the mean of the data produced.

### RNA Isolation and Quantitative RT-PCR

Total RNA was extracted from bacteria grown under different culture conditions using the hot phenol method (Jahn et al., 2008) with some modifications. Briefly, after the lysate was obtained, an equal volume of phenol-saturated water was added, mixed and incubated at 65°C for 5 min. The samples were chilled on ice and centrifuged at 19,000 × *g* for 10 min at 4°C. The aqueous layer was transferred to an 1.5 ml Eppendorf tube, RNA was precipitated with cold ethanol and incubated at -70°C overnight. The RNA was pelleted by centrifugation at 19,000 × *g* for 10 min at 4°C. Pellets were washed with cold 70% ethanol and centrifuged at 19,000 × *g* for 5 min at 4°C. After careful removal of the ethanol, the pellets were air dried for 15 min in the Centrifugal Vacuum Concentrator 5301 (Eppendorf). The pellets were resuspended in 100 μL of DEPC-treated water. Purification of RNA was performed using the TURBO DNA-free kit (Ambion, Inc.). Quality of RNA was assessed using a NanoDrop (ND-1000; Thermo Scientific) and a bleach 2% agarose gel as previously described (Aranda et al., 2012). qRT-PCR was performed as previously reported (Ares et al., 2016). Specific primers were designed with the Primer3Plus software^2^ and are listed in **Table 2**. The absence of contaminating DNA was controlled by lack of amplification products after 35 qPCR cycles using RNA as template. Control reactions with no template (water) and with no reverse transcriptase were run in all experiments. 16S rRNA (HPrrnA16S) was used as a reference gene for normalization and the relative gene expression was calculated using the 2^-ΔΔC_t_^ method (Livak and Schmittgen, 2001). Expression of 16S rRNA remained unaffected in all conditions tested (**Supplementary Figure S1**).

**Table 2:** Primers for qPCR used in this study.

Primers	Sequence	Target gene
rpoD-F	TAT CGC TCA AGT GCC AGA AG	*rpoD*
rpoD-R	TGT TGG GGG CTA GAT CAA AG	
rpoN-F	CAG CGG GTT GAA TAA TGA GG	*rpoN*
rpoN-R	ACG CTT GCG CAC TTT TTC	
fliA-F	TCG TCT AAA AGA GCG CTT GC	*fliA*
fliA-R	CTT CGC ATA CCC CCA AAA AG	
hrcA-F	TTT CTT GCG CAC TGG GTT AC	*hrcA*
hrcA-R	GAA AGA AGC AGC GAT TGA GC	
arsR-F	GAG CGA GTT TTT GCT CCA AC	*arsR*
arsR-R	GCC CGT CTA AAT TAG GCA AAG	
hp0222-F	CTA GGA CGC AAA CCA AAA GC	*hp0222*
hp0222-R	CCC ACG CTT TCT TCT TCT TC	
hp0564-F	GTC GCT GTA GAT GAG CTG AAA C	*hp0564*
hp0564-R	GGC GTT TGA CAA AAG AAT TG	
flgR-F	CAG GCC TTA AAA GTC GCA AG	*flgR*
flgR-R	CGC TAT AAA AGG GTG CTT GG	
hup-F	GTG GAG TTG ATC GGT TTT GG	*hup*
hup-R	TTA GGC ACC CGT TTG TCT TC	
hp1021-F	GTT GCG CAA GAT CCA ATA CC	*hp1021*
hp1021-R	AGG GCG TGT GGA TGA TAA AG	
hspR-F	CGG GCG TGG ATA TTA TCT TG	*hspR*
hspR-R	TGT TTG TGC AGA GCG TCT TG	
fur-F	GAA GAA GTG GTG AGC GTT TTG	*fur*
fur-R	CCT TTT GGC GGA TAG AAT GC	
hsrA-F	GGA AGA AGT CCA TGC GTT TG	*hsrA*
hsrA-R	CAA ACG AGC CTC AAT CCT TG	
cheY-F	TGG AAG CTT GGG AGA AAC TG	*cheY*
cheY-R	CAG AGC GCA CCT TTT TAA CG	
nikR-F	CAT CCG CTT TTC GGT TTC	*nikR*
nikR-R	CAT GTC GCG CAC TAA TTC TG	
crdR-F	CTT AGG CGT GGC TAA AAT GC	*crdR*
crdR-R	CAA ACG CCC CAA AAA CAC	

### Biofilm Formation

*H. pylori* was grown on blood agar medium supplemented with 10% defibrinated sheep blood at 37°C under microaerophilic conditions. Biofilm formation on abiotic surface (polystyrene) was analyzed using 6-well polystyrene plates, inoculated with 3 ml of a bacterial suspension (in BB + FBS, at a final concentration of OD_600nm_ = 0.1) in each well. The plates were incubated during 3 days at 37°C under microaerophilic conditions as previously reported (Cardenas-Mondragon et al., 2016). Supernatant (planktonic) and adhered (sessile) bacteria were recovered for RNA extraction. Fold-change in gene transcription was determined by calculating the relative expression of transcription regulator genes within biofilms (sessile bacteria) as compared to planktonic bacteria. Quantifications were performed in triplicate on three different days and the results shown are the mean of the three experiments.

### Infection of AGS Cells

AGS gastric epithelial cells were grown to about 75% confluence in RPMI-1640 medium containing 10% FBS, and washed thrice with PBS before adding fresh RPMI media with 10% FBS. *H. pylori* 26695 was grown in BB + FBS for 24 h, suspended in RPMI, and added to the AGS cell culture at a multiplicity of infection (MOI) of 100 (bacteria/cell). Infected cells were incubated at 37°C under microaerophilic conditions for 0 or 6 h, and bacteria were recovered. At the end of the incubation period, the *H. pylori*-infected AGS cells were washed thrice with PBS and lysed with 0.1% Triton X-100 for 10 min. Large debris and nuclei were removed by centrifugation for 5 min at 200 × *g* and adhered bacteria were pelleted at 20,000 *g* for 10 min. RNA was extracted from adhered bacteria to determine gene expression. Fold-change in gene transcription was determined by calculating the relative expression of the transcription factors genes with respect to bacteria at time 0 of infection. Fold-change in gene transcription of *H. pylori* grown in RPMI-1640 + FBS (for 0 or 6 h) was calculated as control of expression. Assays were performed in triplicate on three different days and the results shown are the mean of the three experiments.

### Transcription in the Presence of Antibiotics

*H. pylori* was grown in BB + FBS at 37°C for 48 h (stationary phase), with gentle shaking under microaerophilic conditions. The antibiotics kanamycin (Km, 50 μg/mL), chloramphenicol (Cm, 30 μg/mL) or tetracycline (Tc, 10 μg/mL) were added and the cultures were incubated for 1 h as previously described (Christensen-Dalsgaard et al., 2010; Cardenas-Mondragon et al., 2016). Antibiotics were used at the minimal inhibitory concentrations that have been reported for *E. coli* and *S. enterica* (Christensen-Dalsgaard et al., 2010; Maisonneuve et al., 2011; Silva-Herzog et al., 2015; Li et al., 2016). Fold-change in gene transcription was determined by calculating the relative expression of the transcription regulators genes in the presence of each antibiotic as compared to bacteria growing without antibiotics for 1 h. Experiments were performed in triplicate on three different days and the results shown are the mean of the three experiments.

### Heatmap Construction

To show the fold-changes in gene expression, we selected the “heatmap.2” function of the R software, using the “gplots” package. The rows (culturing conditions) were hierarchically clustered (“hclust” function, “ward.D” method) according to the absolute fold-changes in gene expression.

In order to illustrate the presence/absence of transcription factors in all *Helicobacter* genomes, an amino acid sequences content matrix (“heatmap” function) was built using the R software^3^ (v3.2.4). 260 *H. pylori* and 57 *H.* non*-pylori* genomes were retrieved from the NCBI database^4^ by a series of custom Perl scripts. These paired loci were hierarchically clustered (“hclust” function, “ward.D” method) according to their loci-content using a sidelong dendrogram.

### Statistical Analysis

For statistical differences, one-way ANOVA followed by the Tukey’s comparison test was performed using Prism5.0 (GraphPad Software Inc., San Diego, CA, USA). *p* ≤ 0.05 was considered statistically significant.

## Results

### Environmental Cues that Trigger the Expression of Transcription Factor Genes

**FIGURE 1:**
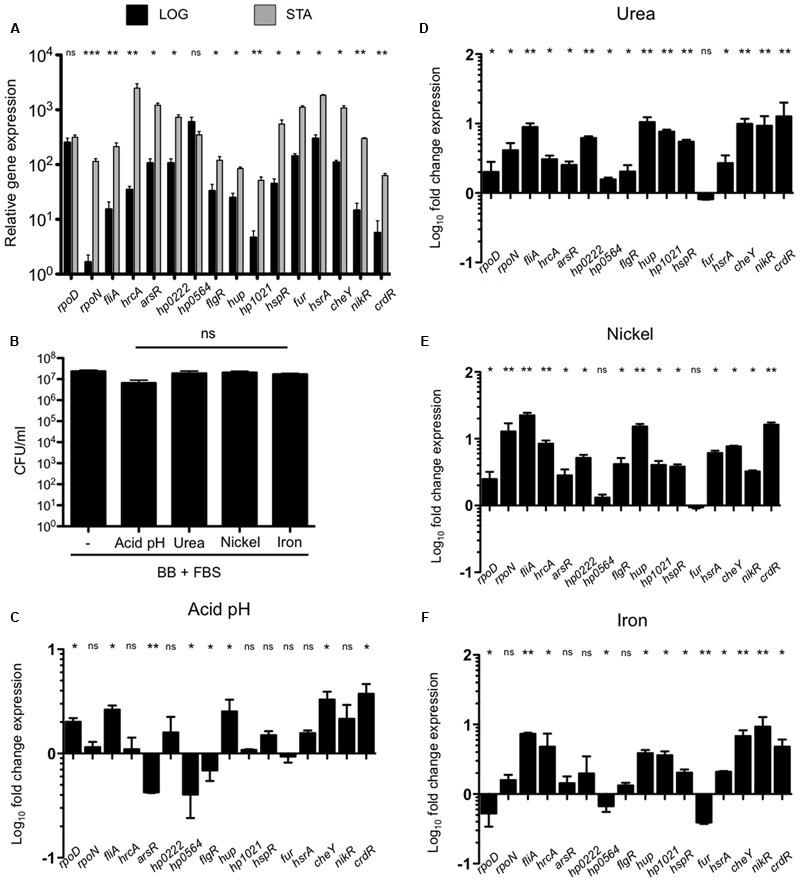
**Effect of environmental cues on expression of transcription factors. (A)** Expression (qRT-PCR) of the transcription factors of *H. pylori* 26695 in exponential (black bars) and stationary growth phase (gray bars). **(B)** Determination of colony forming units (CFU) of *H. pylori* 26695 grown to stationary phase (48 h) in BB + FBS under acid pH (pH 5.5), or in presence of urea (5 mM), nickel (250 μM), or iron (150 μM). Fold-change expression (qRT-PCR) of the transcription factors under acid pH **(C)**, urea **(D)**, nickel **(E)**, and iron **(F)**. Data is represented as fold-change expression of the regulatory gene under different environmental conditions as compared to plain BB + FBS at 48 h. Data represent means and standard deviations of at least three independent experiments. ns, not significant; statistically significant ^∗∗∗^*p* < 0.001, ^∗∗^*p* < 0.01, ^∗^*p* < 0.05.

*H. pylori* adaptation to the gastric mucosa conditions is mediated by a limited number of regulatory genes. An analysis of the reports of 26695 *H. pylori* strain identified 16 genes that code for transcriptional regulators, including three sigma factors (**Table 1**). We performed qRT-PCR on RNA extracted from bacteria grown during both exponential (24 h) and stationary phase (48 h) and the expression of all regulatory genes was calculated during both growth phases. Expression of most genes was higher during stationary phase than in exponential phase, except for *rpoD* and *hp0564* (**Figure 1A**). Therefore, we determined the expression of all transcription regulators during stationary phase in media with acid pH or in the presence of urea, nickel, or iron. None of these environmental variations promoted or inhibited growth of *H. pylori* (**Figure 1B**). Interestingly, the conditions tested resulted mostly in increased expression of transcription regulators (**Figures 1C–F, 4**). Regarding sigma factors, *fliA* expression increased with all treatments, with the highest induction levels observed in response to nickel. The same was true for *rpoD* with exception of treatment with iron, which resulted in down regulation of the gene (**Figure 1F**); whereas *rpoN* expression significantly increased only after exposure to urea or nickel (**Figures 1D,E**). Concerning the other transcriptional regulators, acidic pH resulted in down regulation of *arsR, hp0564*, and *flgR* and a moderate increase of *hup, cheY*, or *crdR*, whereas the remaining genes were unaffected (**Figure 1C**). Exposure of bacteria to urea and nickel ions resulted in more pronounced transcriptional changes (**Figures 1D,E**). However, whereas expression of most transcription factors increased considerably, *hp0564* and *fur* showed only subtle changes in response to urea and nickel. *hp0564, fur*, and *rpoD* were the only genes tested to be down regulated in response to iron, whereas expression of the other transcription regulators increased (*hrcA, hup, hp1021, hsrA, cheY, nikR*, and *crdR*), or did not change (*arsR, hp0222, flgR*, and *hspR*) (**Figure 1F**).

### The Effect of Biofilm Formation and Interaction with Gastric Epithelial Cells on Expression of *H. pylori* Transcription Factors

**FIGURE 2:**
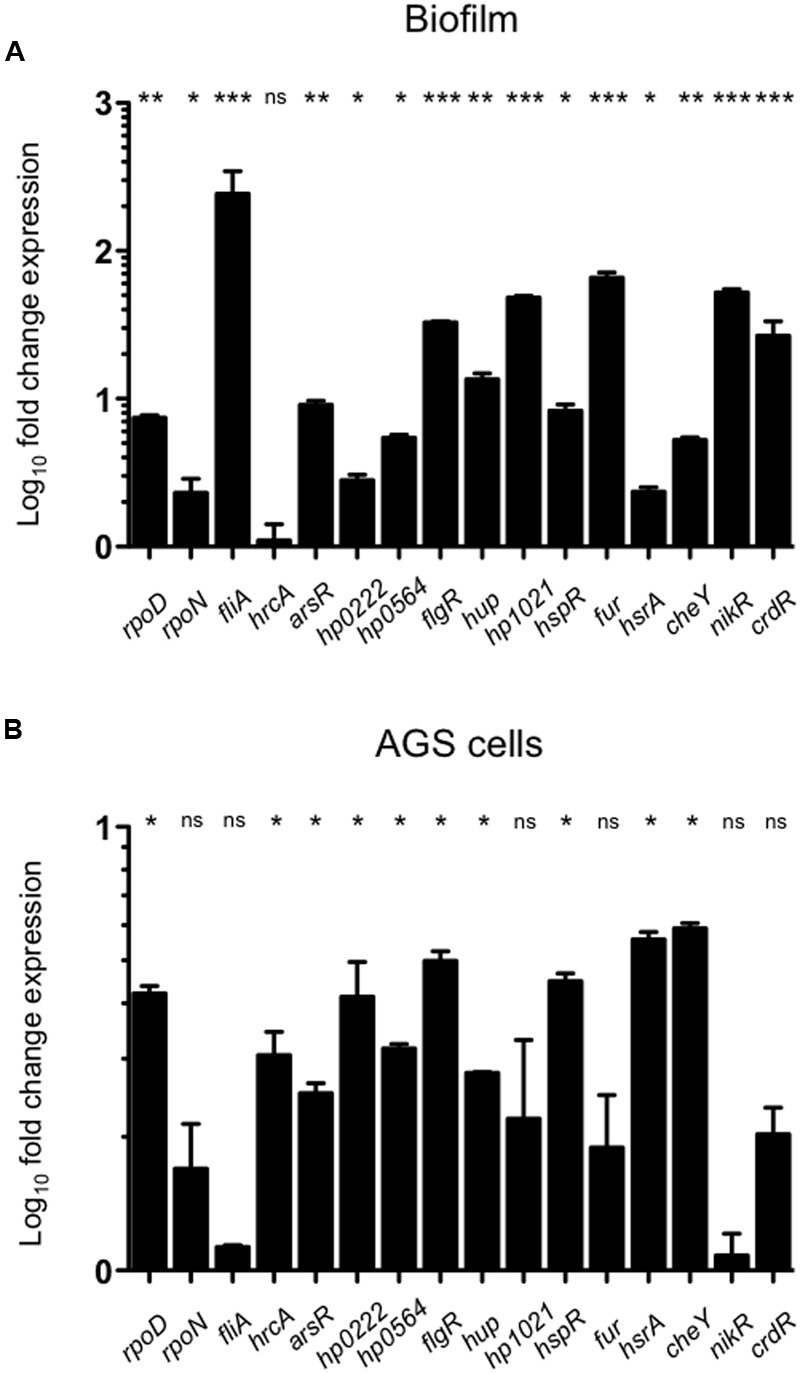
**Expression of transcription regulators during biofilm formation or in response to interaction with AGS cells.** Expression levels of the transcription regulators during bacterial biofilm formation **(A)** or *H. pylori* interaction with AGS cells **(B)** were determined by qRT-PCR. Data are expressed as fold-change expression levels and represent means and standard deviations of at least three independent experiments. ns, not significant; statistically significant ^∗∗∗^*p* < 0.001, ^∗∗^*p* < 0.01, ^∗^*p* < 0.05.

During infection, *H. pylori* closely interacts with the cells of the gastric mucosa, which may result in bacterial biofilm formation in later stages of infection (Carron et al., 2006). To study the effect of bacterial interaction with cells of the gastric mucosa or the growth in biofilms on the expression of transcription regulators, bacteria were grown either stationary on polystyrene surfaces or brought into contact with AGS cells, and their transcription profiles were analyzed. As control for the interaction with AGS cells, *H. pylori* was grown in RPMI-1640 medium for the same amount of time, which did not result in any changes in gene transcription. Expression of *rpoN* did not change during growth in biofilm or upon attachment to AGS cells, whereas *rpoD* expression increased under both conditions (**Figure 2A**). However, the most striking effect among the three sigma factors was a dramatic increase of *fliA* expression in response to biofilm formation (**Figure 2**). Only few of the other regulatory genes remained unaffected by the interaction with abiotic surfaces (*hrcA*), or epithelial cells (*hp1021, fur, nikR*, and *crdR*). All other transcriptional regulators were up regulated upon contact with AGS cells, and to a greater extent during biofilm formation (**Figures 2, 4**).

### Antibiotic Exposure Decreases Expression of Most *H. pylori* Transcription Regulators

**FIGURE 3:**
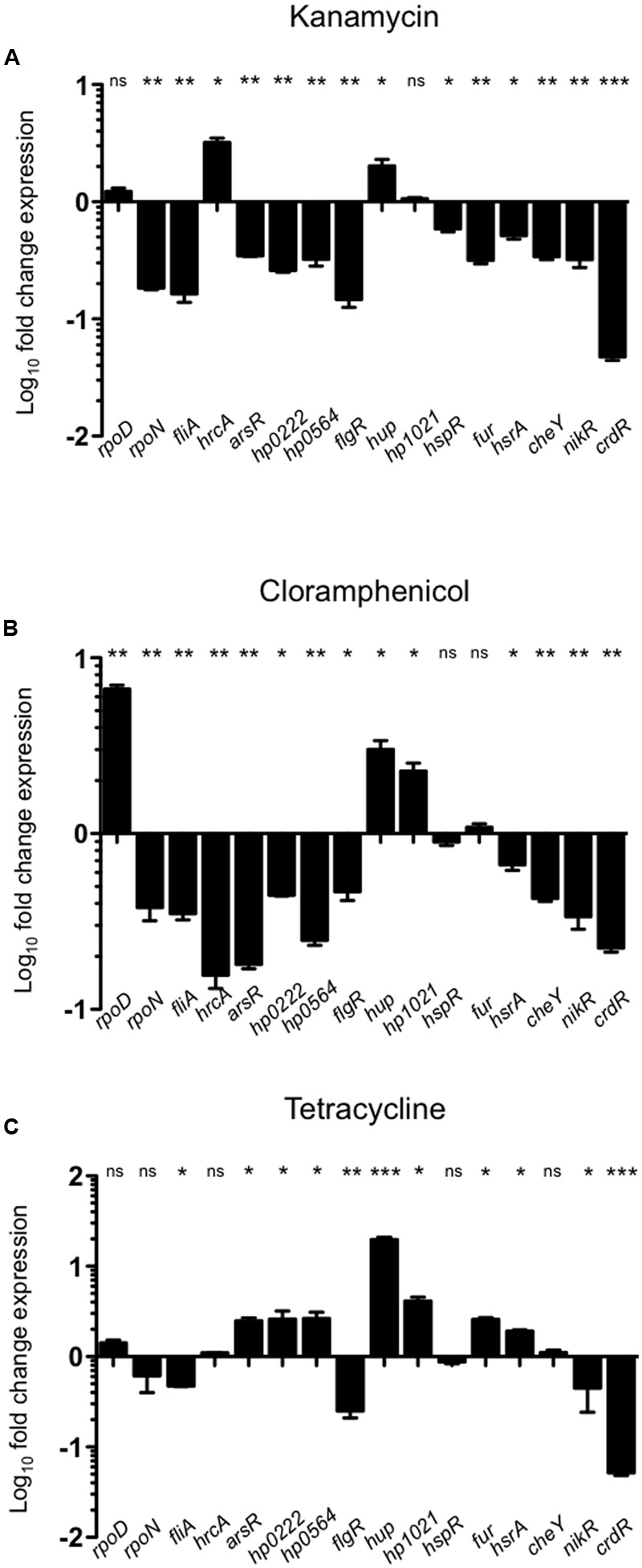
**Effect of antibiotics on expression of transcription factors.** Expression levels of transcription factors after treatment of bacteria with different antibiotics [Kanamycin **(A)**, Chloramphenicol **(B)**, and Tetracycline **(C)**] for 1 h were determined by qRT-PCR and compared to those in untreated bacteria. Data are expressed as fold-change expression and represent the means and standard deviations of at least three independent experiments. ns, not significant; statistically significant ^∗∗∗^*p* < 0.001, ^∗∗^*p* < 0.01, ^∗^*p* < 0.05.

**FIGURE 4:**
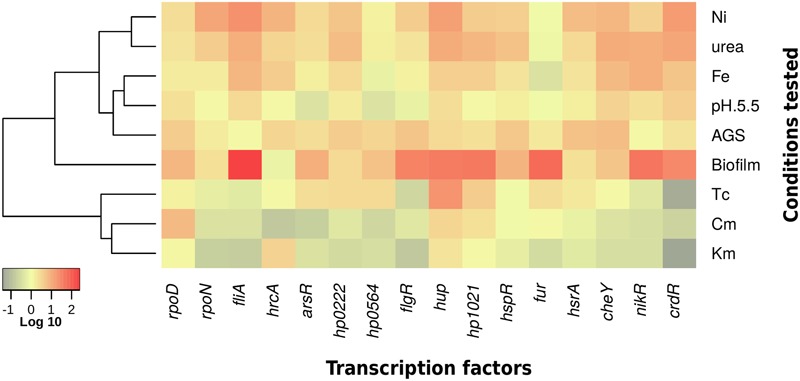
**Summary of effects of different environmental stimuli on expression of *H. pylori* transcription factors.** Heatmap of gene expression levels at diverse culturing conditions. Relative gene expression values are expressed as fold-changes in a Log_10_ scale. The color-coding scale denotes up regulation in red and down regulation in green.

Our group recently reported that antibiotics affect the expression of virulence factors in *H. pylori* (Cardenas-Mondragon et al., 2016). Whereas the environmental conditions tested here mostly up regulated expression of the transcription regulators analyzed, exposure to different antibiotics resulted predominantly in gene repression (**Figures 3, 4**). This was likely not due to compromised cell growth, since the antibiotic concentrations used here did not affect the viability of the bacteria (**Supplementary Figure S2**). Among the three sigma factors, *rpoD* expression was not affected by exposure to kanamycin or tetracycline and increased in response to chloramphenicol (**Figure 3**), whereas expression of *rpoN* and *fliA* was down regulated or not affected after exposure to all three antibiotics tested (**Figure 3**). Expression levels of the other transcription regulators were mostly repressed in response to antibiotic treatment, particularly upon exposure to kanamycin or chloramphenicol (**Figures 3A,B**). Only *hrcA* and *hup* mRNA levels were slightly increased in the presence of kanamycin, whereas those of *hp1021* were not affected (**Figure 3A**). While negatively regulating expression of most transcription factors, chloramphenicol led to a mild increase of *hup* and *hp1021* levels, and did not affect expression of *fur* (**Figure 3B**). In contrast, tetracycline had stimulating effects on the expression of several transcription factors, including *hp0166, hp0222 hp0564, hup*, and *hp1021* (**Figure 3C**). Transcription of *flgR, nikR*, and *crdR* decreased upon tetracycline treatment, whereas transcription levels of *hrcA, hspR*, and *cheY* were not affected (**Figure 3C**).

### Transcriptional Regulator Genes Are Highly Conserved in *H. pylori* Strains

**FIGURE 5:**
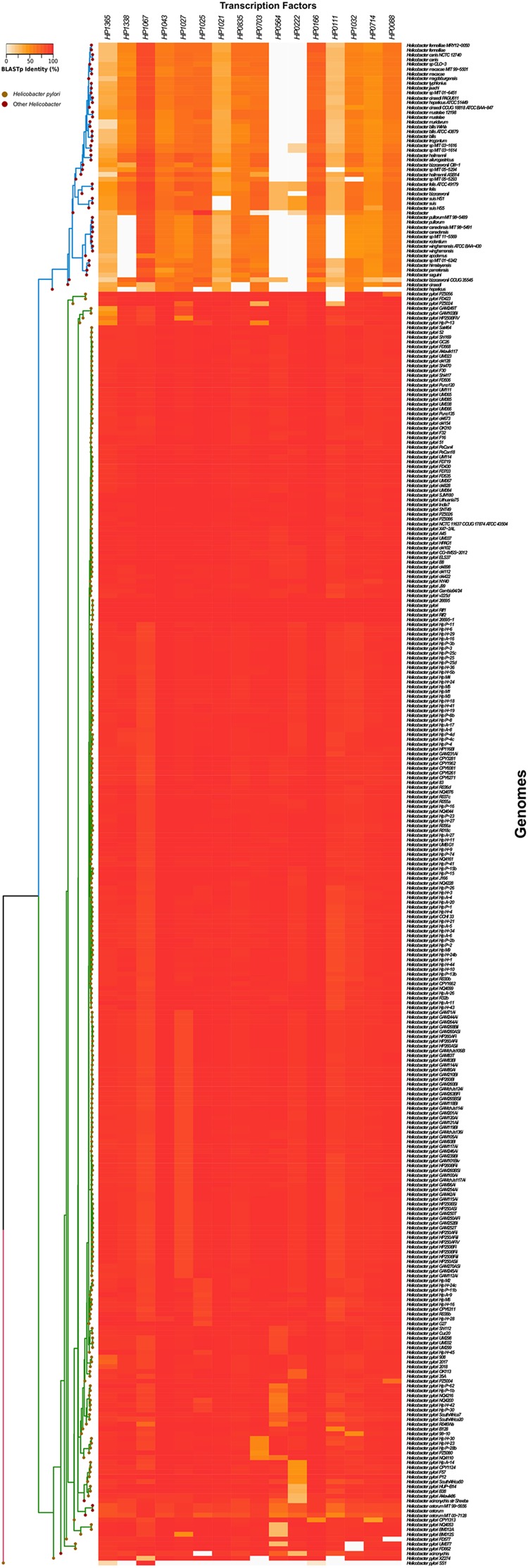
**Prevalence of transcription regulators in the *Helicobacter* genus.** Amino acid sequences of *Helicobacter* transcription factors were analyzed. Heatmaps and hierarchical clustering of selected amino acid sequences for transcription factors for both *Helicobacter* and *Helicobacter* non-*pylori* showing the identity of transcription regulators were created using the R (version 3.2.4) hclust function with the “ward.D” method.

We performed a Blast search in genomes deposited in GenBank^5^ using the amino acids sequences of the 16 transcriptional regulators identified in *H. pylori* 26695. The transcription factors were highly prevalent and well conserved among different *H. pylori* isolates (**Figure 5**). We studied the occurrence of these genes in other *Helicobacter* species, and found that their presence changed from one species to another. Two species that are phylogenetically closely related to *H. pylori, H. acinonychis* (isolated from big cats), and *H. cetorum* (isolated from marine mammals), encoded all 16 transcriptional regulators with high identities to those of *H. pylori* strains, and clustered closer to the *H. pylori* strains (**Figure 5**). Interestingly, transcription regulators such as *hp0222* and *hp0564* presented low prevalence in most of the *H*. non-*pylori* strains, showing that both proteins are highly conserved in *H*. *pylori, H*. *acinonychis*, and *H*. *cetorum*.

## Discussion

*H. pylori* is a highly specialized bacterium that is exclusively found in the human gastric mucosa. In this work, we describe the expression of *H. pylori* transcriptional regulatory genes under different environmental conditions. Most transcription factors were highly expressed when *H. pylori* reached the early stationary phase. At this growth phase, *H. pylori* is exposed to specific stress signals such as pH changes, starvation, reactive oxygen species that would activate its transcriptional repertoire, suggesting that the stationary phase may mimic the conditions found by the bacteria in the host. Acid pH, the presence of urea, nickel, or iron are environmental cues required for optimal adaptation of *H. pylori* to its natural niche. Whereas all of the above conditions boosted *fliA* expression, *rpoD* showed only a mild increase in transcription after bacterial exposure to acid pH, urea, and nickel, and a decrease in response to iron. The expression of *rpoN* significantly increased upon treatment of bacteria with urea or nickel; RpoN was initially described as regulator of flagellar genes, but recent studies show that it also controls several bacterial regulatory processes involved in energy metabolism, biosynthesis, protein fate, oxidative stress, and virulence (Sun et al., 2013). *fliA* was strongly expressed even under environmental conditions that are not common inducers of flagella synthesis, but are known to affect other bacterial components or pathways. In fact, FliA regulates expression of outer membrane proteins, lipopolysaccharide synthesis, DNA restriction, and CagA (Josenhans et al., 2002; Baidya et al., 2015), a protein involved in virulence and associated with the development of gastric carcinoma (Ohnishi et al., 2008). Our data support the notion that FliA regulates different bacterial functions other than the flagellum.

*H. pylori* is exposed to changes in pH while passing from the stomach lumen through the mucus layer to interact with epithelial cells, and this pH gradient is used by the bacteria for spatial orientation (Schreiber et al., 2004). Accordingly, changes in pH strongly affect expression of transcriptional regulators that control genes involved in colonization and persistence in the human host. Our data show that an acidic pH repressed *arsR, hp0564*, and *flgR*, while stimulating *hup, cheY*, and *crdR*. The *hup* gene codes for the HU nucleoid protein, which has a regulatory role in the response to acid stress in *H. pylori*. Thus, *hup* mutants are less viable than wild type bacteria at pH 5.5 and during stomach colonization due to down regulation of both urease (*ureA*) and arginine decarboxylase (*speA*) in the absence of HU nucleoid protein (Wang et al., 2012; Almarza et al., 2015). CheY and CrdR response regulators are also crucial for a successful colonization of the animal stomach (Foynes et al., 2000; Panthel et al., 2003; McGee et al., 2005; Terry et al., 2005). CheY expression is essential for the chemotactic motility required to reach and colonize the gastric epithelia, and is likely to be triggered in the acidic milieu of the stomach lumen. In contrast, CrdR has not been shown to be involved in the regulation of gene expression in response to acidic pH (Pflock et al., 2007b), although copper can be present in the acidic environment of the human stomach, and regulation of its uptake is important for keeping the balance between supplying copper as respiration co-factor, and avoiding copper-induced toxicity (Haley and Gaddy, 2015). Interestingly, the master regulator of the acid response *arsR* was repressed in acidic pH, which confirms reports that ArsR may also act as transcriptional auto-repressor under an acidic pH (Dietz et al., 2002).

Urea, nickel, and iron are crucial for *H. pylori* pathogenesis and they control regulatory networks responding to their presence. We found that expression of most of the transcription regulators tested increased when bacteria were exposed to urea, nickel, or iron. Nickel serves as essential co-factor for the urease enzyme, which enables *H. pylori* survival at acidic pH (Khan et al., 2009). The up regulation of *nikR* expression that we observed contrasted with the auto-negative regulation reported for the *nikR* promoter (Delany et al., 2002; Contreras et al., 2003). The conditions of growth (stationary phase) and nickel concentrations (250 μM) that we tested resulted in increased *nikR* expression. However, *nikR* expression showed slight variations in response to low (1 μM) and high (100 μM) concentrations of nickel (Davis et al., 2006), suggesting that nickel may modulate *nikR* transcription in a concentration-dependent manner. Interestingly, *rpoD* and *fur* were down regulated in the presence of iron. While iron-mediated *fur* repression can be explained by the negative auto-regulation of this transcription factor upon iron-binding (Delany et al., 2002), the decrease in *rpoD* levels is hard to explain. Whilst the evaluation of each environmental condition provides relevant information about *H. pylori* physiology, the combination of these stimuli could better mimic the *in vivo* response of the bacteria in the infection context.

One of the strategies employed by *H. pylori* to persist and colonize the stomach is biofilm formation. Analysis of bacteria grown in biofilm revealed an interesting expression pattern of the three sigma factors: whereas *rpoN* was not affected, expression of *rpoD* and *fliA* increased during biofilm formation. FliA has been found to control the *lpxC* gene, which is involved in the early steps of lipid A synthesis in *H. pylori* (Josenhans et al., 2002). The marked increase of *fliA* expression that we found in sessile, aggregated bacteria is in agreement with reports about the effect of lipid A architecture on biofilm formation (Gaddy et al., 2015). In addition, with the exception of *hrcA*, expression of all transcription factor genes studied increased during biofilm formation, and the relative increase of several of them was the highest increase observed across all the conditions tested. This remarkably activated state of the regulatory transcriptome highlights the importance of forming sessile microbial communities in *H. pylori* ecology.

Similar to the response during biofilm formation, the presence of gastric epithelial cells significantly increased expression levels of several transcription factors, except for *fur, nikR*, and *crdR*. Expression of these three master regulators of virulence remained unaffected in our AGS cell model, which correlates with the previously reported lack of activation or repression of these regulators after the interaction with gastric epithelial cells (Kim et al., 2004).

It has been hypothesized that the reduced number of transcriptional regulators in the *H. pylori* genome has been compensated by gain of functions in the remaining transcription factors, as compared to their functions by homologs found in other species (Ernst et al., 2005a). For instance, the *H. pylori* Fur protein was not only found to be involved in iron homeostasis, but it also participated in several other additional pathways including those of oxidative stress resistance (Harris et al., 2002) and acid regulation (Bijlsma et al., 2002; van Vliet et al., 2004), and has been found essential for colonization of the gastric mucosa (Bury-Mone et al., 2004). Moreover, unlike Fur homologs in other species, *H. pylori* Fur has been found to mediate gene regulation even in its iron-free (apo) form (Bereswill et al., 2000; Delany et al., 2001; Carpenter et al., 2013). Interestingly, whereas most conditions tested here showed only moderate effects on Fur expression, biofilm formation resulted in a marked up regulation of the gene, suggesting functions beyond regulation of iron metabolism.

The presence of antibiotics can alter the expression of genes related to the bacterial stress and virulence on a transcriptional level. Interestingly, most regulatory genes were repressed in response to antibiotic treatment. *rpoN, fliA, flgR*, and *crdR* genes presented a negative regulation profile in the presence of kanamycin, chloramphenicol, and tetracycline. In contrast, expression levels of *rpoD* and *hup* were highly stimulated under chloramphenicol or tetracycline treatment. These last antibiotics inhibit bacterial translation, differentially affecting the 50S and 30S ribosomal subunits, respectively. The molecular mechanisms responsible for the regulation in expression of transcriptional regulators in the presence of antibiotics have been poorly studied. About this, 16S rRNA expression was affected in the presence of kanamycin and chloramphenicol, showing that this gene was not completely stable and that antibiotic treatment may have affected the expression of this reference gene under these conditions. For a better analysis in presence of these antibiotics, it is necessary the selection and validation of other reference genes for qRT-PCR normalization as was recently reported (Martins et al., 2017).

During analysis of *Helicobacter* sequences we found that the transcriptional regulators were highly identical among *H. pylori* species. Interestingly, *H. acynonichis* and *H. cetorum* grouped together with *H. pylori*, corroborating the close phylogenetic relation between these species. The transcriptional regulators HP0222 and HP0564 appear to be conserved in *H. pylori* and its closely related species, while they were absent in most of the remaining *Helicobacter* species. Since *H. pylori, H. acynonichis*, and *H. cetorum* are all found within mammalian stomachs, these two regulators may confer an adaptive advantage in this particular ecological niche. In line with these findings, expression levels of both, *hp0564* and *hp0222* increased in contact with AGS gastric epithelial cells, corroborating a report by Kim et al. (2004) on *hp0222*. However, we did not observe enhanced *hp0222* expression under acidic pH, contrasting with the report by Ang et al. (2001).

Recently, we reported the transcriptional profiling of type II toxin-antitoxin genes under different environmental conditions (Cardenas-Mondragon et al., 2016). The type II antitoxins function as transcriptional repressors of their own expressions (Yamaguchi and Inouye, 2011) and also regulate the expression of other genes related with cellular functions such as biofilm formation, persistence, and the general stress response (Wang and Wood, 2011; Hu et al., 2012). Our findings here expand the transcriptional repertoire of *H. pylori* to respond to the different stresses found in the stomach niche.

In summary, our data show that the repertoire of transcriptional regulators of *H. pylori* possesses a functional plasticity needed to respond to different environmental cues and to integrate them for the survival and persistence of this bacterium in the stomach niche.

## Author Contributions

Conceived and designed the experiments: MDC. Performed the experiments: MDC, KvB, MA, LP, JM-C, HV-S, and CJ-G. Analyzed the data: MDC, KvB, and MA. Wrote the paper: MDC, KvB, and JT.

## Conflict of Interest Statement

The authors declare that the research was conducted in the absence of any commercial or financial relationships that could be construed as a potential conflict of interest.

## Footnotes

1 ftp://ftp.ncbi.nlm.nih.gov/genomes/genbank/bacteria/

2 http://www.bioinformatics.nl/cgi-bin/primer3plus/primer3plus.cgi/

3 http://www.R-project.org/

4 ftp://ftp.ncbi.nlm.nih.gov/genomes/genbank/bacteria/

5 http://www.ncbi.nlm.nih.gov/genbank/

## Supplementary Material

FIGURE S1 **Expression of reference gene (HPrrnA16S) under different environmental conditions.** Panels show the expression of reference gene during stationary phase in BB + FBS with changes in pH and concentrations of urea, nickel, and iron **(A)**, or in presence of antibiotics **(B)** or in contact on abiotic and biotic surfaces **(C)**. (-) Indicates the BB + FBS plain (neutral pH with no addition of components). Quantification of expression is showed as copies of HPrrnA16S/μg RNA.

FIGURE S2 **Effect of antibiotics on *H. pylori* growth.** Determination of colony forming units (CFU) of *H. pylori* 26695 grown during 1 h in presence of antibiotics (Km, Kanamycin; Chloramphenicol, Cm; Tetracycline, Tc).
