# Supplementary figures and images for: Gene Expression Profiling of Transcription Factors of Helicobacter pylori under Different Environmental Conditions
Source: Front Microbiol. 2017 Apr 10;8:615. doi: 10.3389/fmicb.2017.00615 (PMC5385360; doi:10.3389/fmicb.2017.00615)

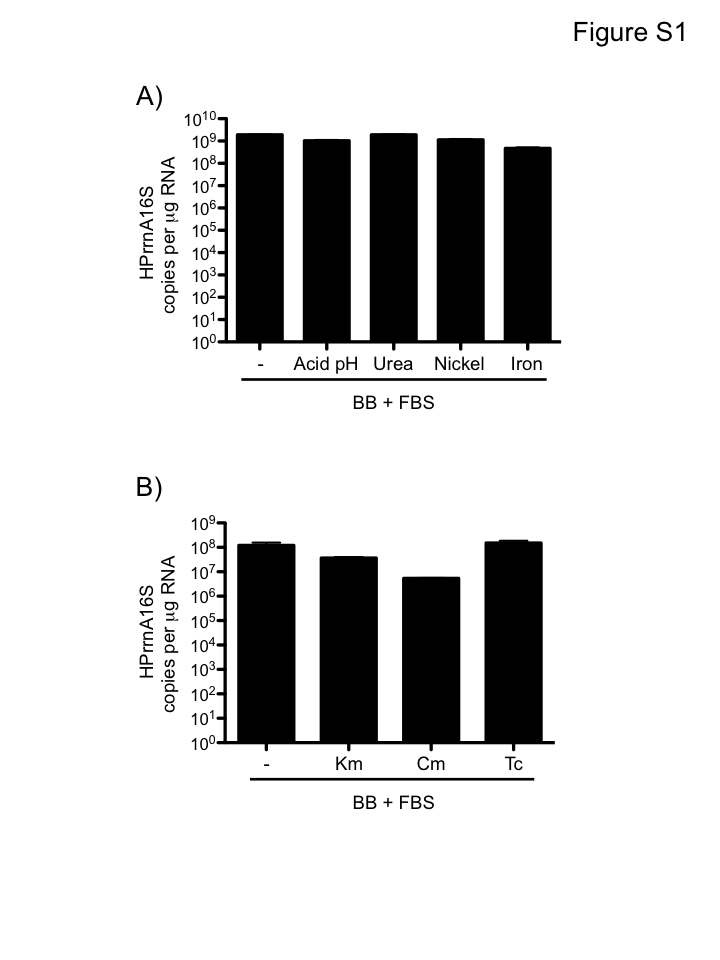

Supplement: FIGURE S1 — Expression of reference gene (HPrrnA16S) under different environmental conditions. Panels show the expression of reference gene during stationary phase in BB + FBS with changes in pH and concentrations of urea, nickel, and iron (A), or in presence of antibiotics (B) or in contact on abiotic and biotic surfaces (C). (-) Indicates the BB + FBS plain (neutral pH with no addition of components). Quantification of expression is showed as copies of HPrrnA16S/μg RNA. [file Image_1.TIFF]

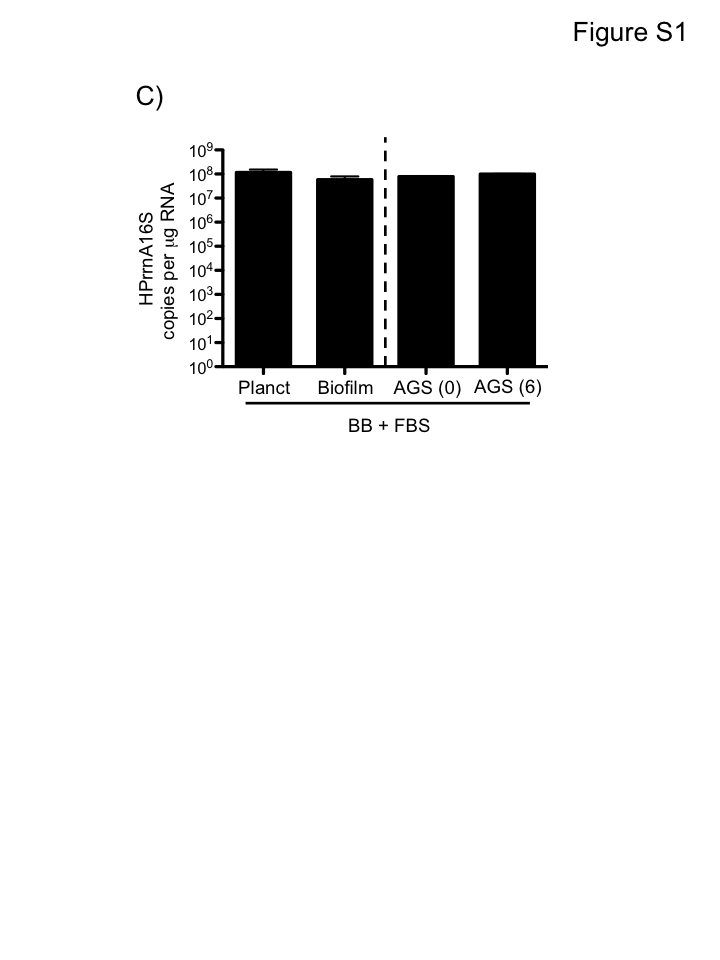

Supplement: FIGURE S2 — Effect of antibiotics on H. pylori growth. Determination of colony forming units (CFU) of H. pylori 26695 grown during 1 h in presence of antibiotics (Km, Kanamycin; Chloramphenicol, Cm; Tetracycline, Tc). [file Image_2.TIFF]

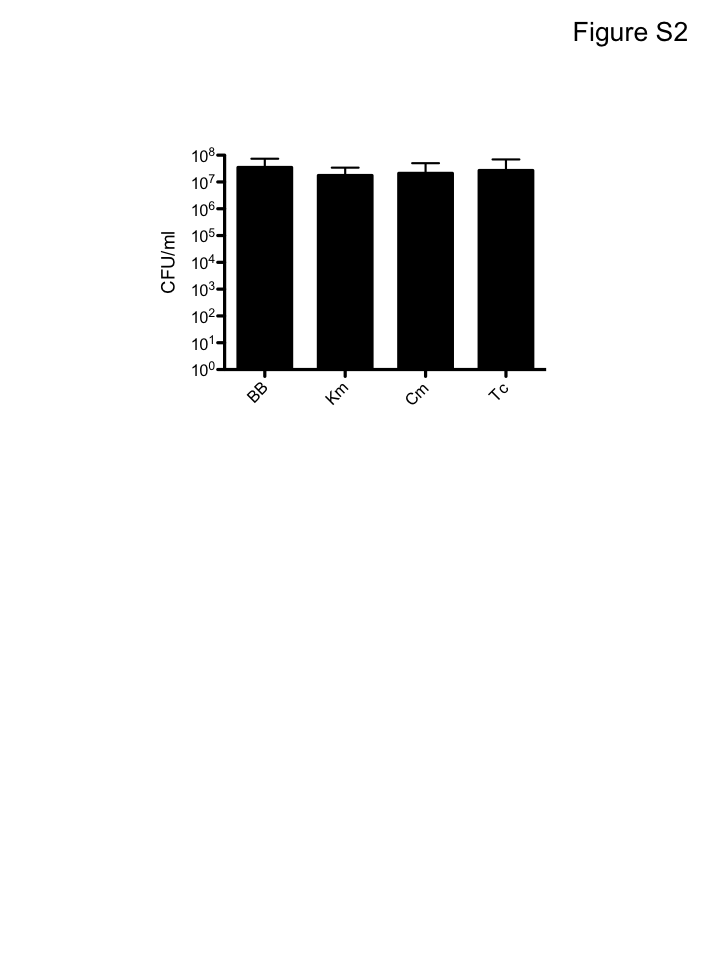

Supplement: Supplementary file 3 [file Image_3.TIFF]
